# Development of a Bioluminescent BRCA1-Deficient Xenograft Model of Disseminated, High-Grade Serous Ovarian Cancer

**DOI:** 10.3390/ijms20102498

**Published:** 2019-05-21

**Authors:** Yen Ting Shen, Lucy Wang, James C. Evans, Christine Allen, Micheline Piquette-Miller

**Affiliations:** Department of Pharmaceutical Sciences, Leslie Dan Faculty of Pharmacy, University of Toronto, Toronto, ON M5S 3M2, Canada; russell.shen@mail.utoronto.ca (Y.T.S.); lucille.wang@mail.utoronto.ca (L.W.); james.evans@utoronto.ca (J.C.E.); cj.allen@utoronto.ca (C.A.)

**Keywords:** BRCA-deficiency, bioluminescent imaging, ovarian cancer, xenograft, metastasis, ascites, intraperitoneal, carboplatin, high-grade serous

## Abstract

Successful translation of preclinical data relies on valid and comprehensive animal models. While high-grade serous ovarian cancer (HGSOC) is the most prevalent subtype, the most commonly used ovarian cancer cell lines are not representative of HGSOC. In addition, 50% of ovarian cancer patients present with dysfunctional *BRCA1/2*, however currently there is a shortage of BRCA-deficient models. By utilizing the OVCAR8 cell line, which contains a hypermethylated *BRCA1* promoter, the aim of the current study was to establish and characterize an animal model for BRCA-deficient HGSOC. Transfection of the luciferase gene to OVCAR8 cells enabled bioluminescent imaging for real-time, non-invasive monitoring of tumor growth. The resulting model was characterized by peritoneal metastasis and ascites formation at late stages of disease. Immunohistochemical staining revealed high-grade serous histology in all resected tumor nodules. Immunoblotting and qPCR analysis demonstrated BRCA1 deficiency was maintained in vivo. Moderate to strong correlations were observed between bioluminescent signal and tumor weight. Lastly, intraperitoneal administration of carboplatin significantly reduced tumor growth as measured by bioluminescence. The current model demonstrated BRCA1 deficiency and a high resemblance of the clinical features of HGSOC. This model may be well-suited for evaluation of therapeutic efficacy in BRCA-deficient HGSOC.

## 1. Introduction

Ovarian cancer accounts for the majority of gynecological malignancy-associated deaths in North America. It is estimated that, out of the 200,000 new diagnoses in 2018, over half of the patients will eventually die from the disease [1]. Early diagnosis for ovarian cancer is clinically challenging due to a lack of obvious symptoms at early stages of disease [2]. Consequently, patients often present at an advanced stage, at which point the rate of survival is low [3]. The most prevalent and aggressive subtype of ovarian cancer is of epithelial origin and with high-grade serous histology [4,5]. While the majority of patients achieve clinical remission following first-line chemotherapy, most relapse with platinum resistant diseases [6]. It is clear that there is an urgent need for better and more effective treatments. However, only 5% of new antitumor agents reach phase III clinical trials largely due to a lack of preclinical models resembling the human disease [7].

To translate preclinical findings, well-characterized models that recapitulate human ovarian cancer (both clinically and genetically) are required. While both genetically engineered mouse [8] and patient-derived tumor models [9,10,11] generate histologically relevant tumor microenvironments, these models can be technically challenging and costly. In contrast, models developed from human ovarian cancer cell lines provide a relatively simple and economical approach. Multiple cell lines with various histological representations have been shown to be tumorigenic in mice [12]. However, recent analyses of these cell lines demonstrate genetic and histological deviations from that which is seen clinically [13,14]. Furthermore, despite advances in the field, there are very few xenograft models that harbor *BRCA* mutations [12]. BRCA1/2 proteins play an important role in homologous recombination. Mutations in the *BRCA* gene, which occur in up to 20% of ovarian cancer patients [15], leads to dysfunctional DNA double-strand break repair. Moreover, epigenetic silencing of the *BRCA* genes or genetic alteration in other proteins of the homologous recombination pathway is commonly observed. Together, these changes are collectively termed “BRCAness” [16]. BRCA deficiency is frequently observed in HGSOC and therefore is an important factor in predicting treatment outcome and prognosis [17]. *BRCA*-deficient cells are more susceptible to platinum-induced DNA damage [18], and *BRCA*-deficient patients have shown higher sensitivity to platinum-based chemotherapy [19,20]. Although *BRCA1* germline deficiency is associated with higher initial platinum sensitivity, patients presenting with somatic *BRCA1* mutations and the BRCAness phenotype have the poorest overall survival [15]. With the emerging clinical importance of BRCA mutations and BRCAness, xenograft models with non-functional BRCA are in need.

Due to the increasing utilization of intraperitoneal or orthotopic models of cancer in preclinical studies, bioluminescence imaging (BLI) is increasingly utilized as a means to monitor disease progression. BLI is a technique that utilizes light produced from the catalytic reaction of luciferin by the luciferase enzyme [21,22]. BLI signals correlate with the number of tumor cells both in vitro and in vivo [23,24] with high sensitivity, making BLI ideal for tracking metastasis [25]. Importantly, BLI allows continuous monitoring without the need for animal sacrifice. Therefore, each animal can act as its own control, reducing variability and bias when assessing treatment response.

In the present study, we developed a clinically relevant model of HGSOC with BRCA1 deficiency. Clinically relevant tumor parameters were evaluated by post-mortem examination and collected tumor samples were further characterized by immunohistochemistry, immunoblotting, and methylation-specific q-PCR. Finally, the validity of the model for assessment of chemotherapeutic efficacy was demonstrated by monitoring tumor burden following treatment with carboplatin.

## 2. Results

### 2.1. Real-time Monitoring of OVCAR8^luc^ Tumor by Bioluminescence Imaging

**Figure 1:**
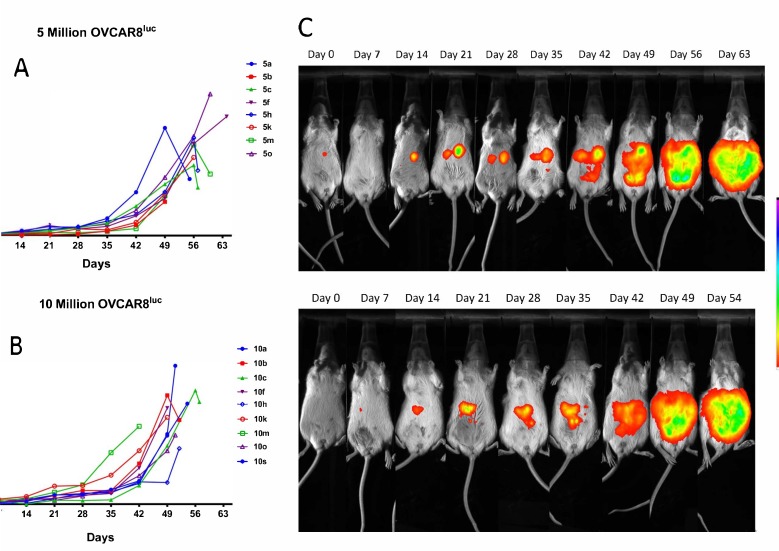
Quantification of bioluminescent signal in mice inoculated with (**A**) 5 million and (**B**) 10 million OVCAR8^luc^ cells. Bioluminescent imaging (BLI) was performed as described in methods; each line represents a single mouse. (**C**) The representative figures show successive images of the same animal in each group, top: 5 million group; bottom: 10 million group. Color scale bar indicates photon/s/mm^2^ of the camera field.

Following tumor inoculation with either 5 × 10^6^ or 10 × 10^6^ cells, mice maintained a body condition score of 3 until week 7 when they became increasingly ill until endpoint. Ascites fluid production was observed near the study endpoint as demonstrated by a rapid increase in abdominal girth from 49 days post inoculation (Figure S1). At endpoint, the mean ascites volumes were 4.56 mL and 4.50 mL for the 5 and 10 million groups, respectively. Although tumor nodules were not palpable throughout the study, mice that were successfully inoculated displayed detectable, bioluminescent tumor nodules at 14 days post-injection in both groups (Figure 1). As anticipated, the mean bioluminescent signal in the 10 million group (6.06 × 10^8^ p/s) was approximately 1.5-fold higher as compared to the 5 million group (4.35 × 10^8^ p/s), suggesting a greater tumor burden. Consequently, mice injected with 5 million OVCAR8^luc^ cells exhibited significantly longer median survival times than mice injected with 10 million cells (57 days versus 51 days respectively) (Figure S2). However, the changes in BLI signaling over time, which reflects the tumor growth rate, did not significantly differ between groups (Figure S3).

### 2.2. OVCAR8^luc^ Model Recapitulate Clinical Features of Ovarian Cancer

**Figure 2:**
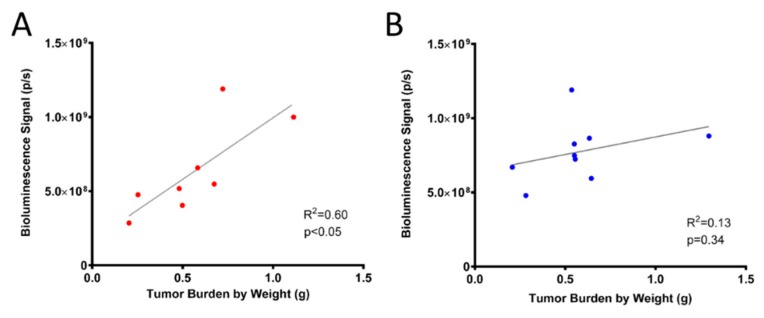
Correlation of bioluminescent signal with tumor weight for (**A**) mice inoculated with 5 million cells (*n* = 8) and (**B**) mice inoculated with 10 million cells (*n* = 9).

At the end of the study, tumor nodules were collected, weighed, and measured for the determination of tumor volume. Tumor weight and volume were subsequently correlated with the in vivo bioluminescence signal using Pearson’s correlation coefficient. Correlations of *R*^2^ = 0.60 and 0.13 were observed between bioluminescence and tumor weight for the 5 million and 10 million groups, respectively (Figure 2A,B).

**Figure 3:**
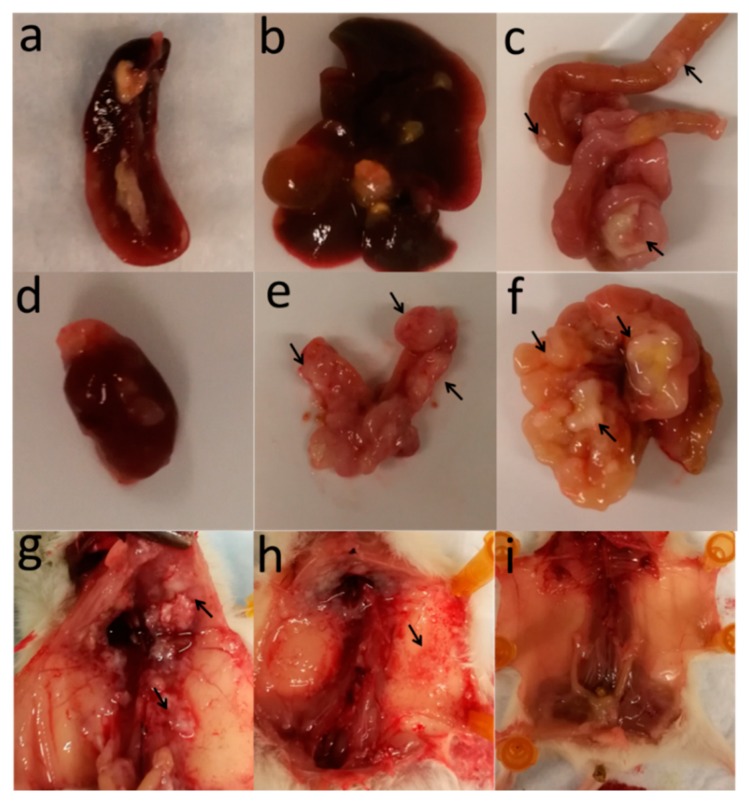
Intraperitoneal OVCAR8^luc^ model produced disseminated disease with tumor nodules in multiple organs including: (**a**) spleen; (**b**) liver; (**c**) small intestine; (**d**) kidney; (**e**) ovaries; (**f**) omentum; (**g**) diaphragm; and (**h**) peritoneal wall; (**i**) normal peritoneal cavity.

Following post-mortem examination, all mice sacrificed at endpoint presented with disseminated disease as evident by multiple tumor nodules throughout the peritoneal cavity and invasion into the peritoneal wall (Figure 3). Metastatic tumors were also observed on the diaphragm, liver, intestine and kidneys, a clinical feature often seen in ovarian cancer patients.

**Figure 4:**
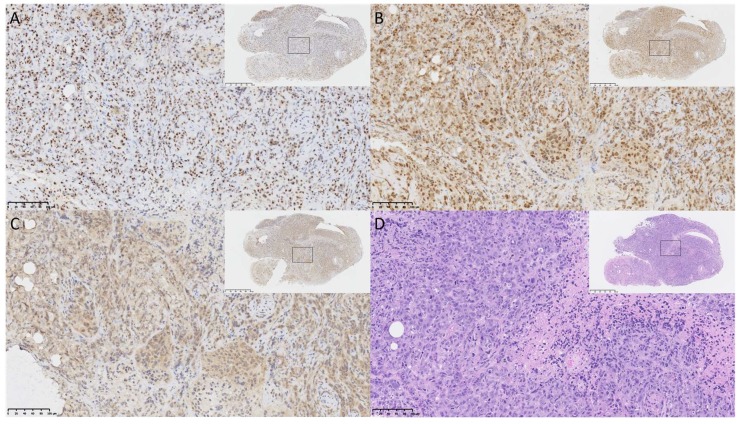
Immunohistology of OVCAR8^luc^ xenografts, using antibodies specific for (**A**) WT1; (**B**) PAX8; (**C**) HIF1-α or (**D**) after staining with H&E. Xenografts were obtained from mice at 56 days post-inoculation of 5 million cells. Slides were deparaffiniated and stained with appropriate antibodies or stain as described in the methods. Figures show representative images of tumor sections collected from mouse 5k at 20× magnification, scale bar equal to 100 µm. Inserts show 4× magnification of the same tumor section, scale bar equal to 500 µm.

To validate the histology of the OVCAR8^luc^ xenograft, tumor samples were fixed and subsequently paraffin embedded for immunostaining of the defined serous histology markers PAX8 and WT1. As shown in Figure 4, strong staining was observed for both markers (Figure 4A,B), suggesting that the current model recapitulates this aspect of human disease. In addition, hypoxia and necrosis were observed in the tumor core, as shown by consistent staining of HIF-1α and compacted nuclei with leakage of cytoplasmic content (Figure 4C,D and Figure S4).

### 2.3. OVCAR8^luc^ Tumor Model is BRCA1 Deficient

**Figure 5:**
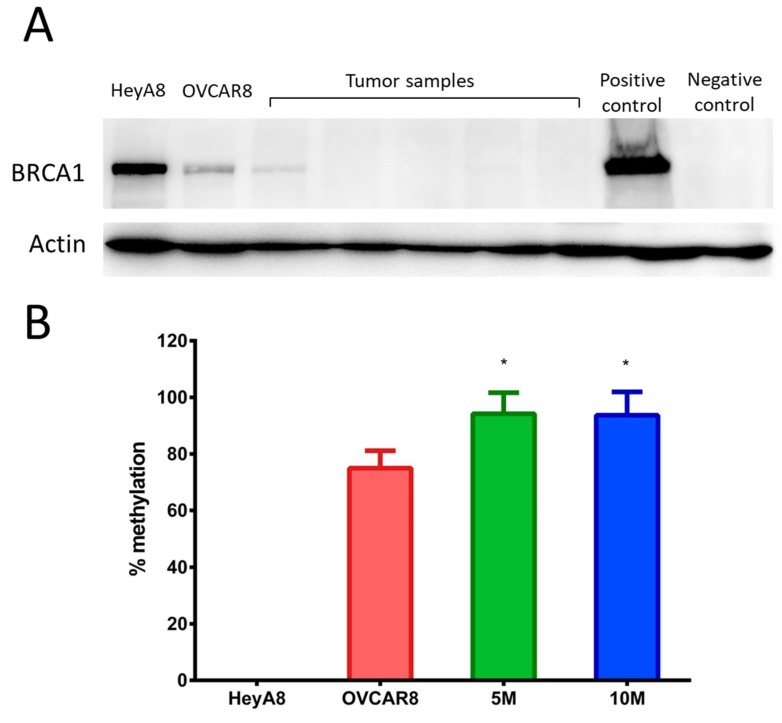
BRCA1 (**A**) expression and (**B**) methylation in BRCA1 proficient HEYA8 cells and BRCA1 deficient OVCAR8^luc^ cells and xenografts. BRCA1 expression as measured by western blot analysis. The extent of *BRCA1* promoter methylation was examined by methylation-specific PCR in HEYA8 and OVCAR8 cells compared to tissue lysate obtained from tumor samples in mice inoculated with 5 or 10 million OVCAR8^luc^ cells. Data are shown as mean ± SD from three independent experiments. Statistical analysis was done by one-way ANOVA with Bonferroni’s test, * *p* < 0.05, compared to OVCAR8.

Reduced BRCA1 expression was confirmed in OVCAR8 cells by comparing BRCA1 protein expression to BRCA proficient HEYA8 cells. Indeed, as shown in Figure 5A, BRCA1 expression for OVCAR8 cells in culture was less abundant as compared to HEYA8. Importantly, low or undetectable BRCA1 expression was observed across all tumor samples, suggesting BRCA deficiency was retained in our in vivo model. Furthermore, western blots revealed that BRCA1 expression in tumor samples was less than that of OVCAR8 cells grown in culture. To determine whether the decreased expression of BRCA1 was due to persistent methylation, we evaluated the degree of methylation at the *BRCA1* promoter region in all tumor samples. As expected, no detectable methylation was observed for BRCA1 wild type HEYA8 cells. Conversely, over 70% methylation was seen in the cultured OVCAR8 cells, which is consistent with reports in the literature (Figure 5B). Interestingly, all tumor samples exhibited higher degrees of methylation than cells grown in culture, as demonstrated by over 80% methylation of the promoter region. These results suggest that *BRCA1* epigenetic changes in OVCAR8 cells are maintained in vivo during the development of peritoneal xenografts.

### 2.4. Efficacy of Carboplatin in OVCAR8 Xenograft Model

**Figure 6:**
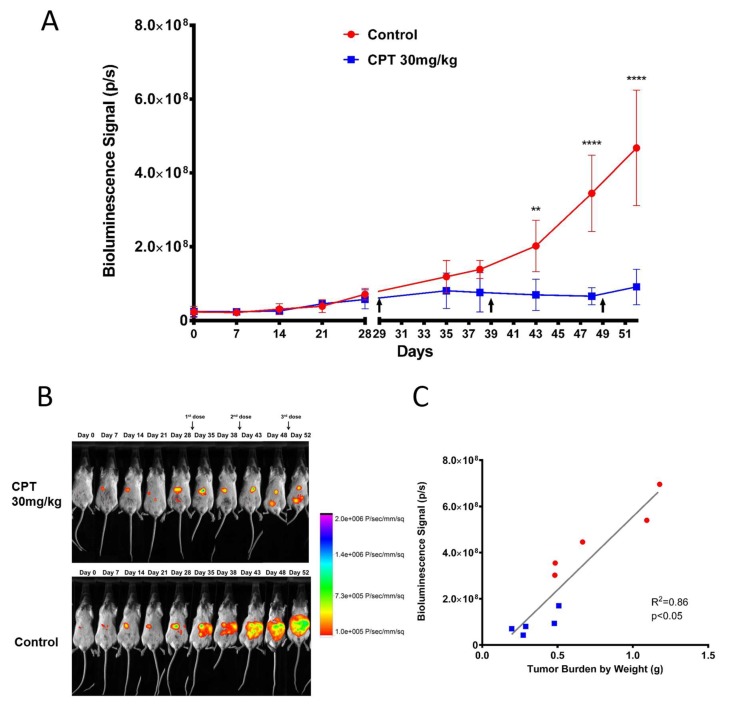
Effect of carboplatin on the growth of OVCAR8^luc^ xenografts in response to intraperitoneal carboplatin (30 mg/kg). Treatment was initiated at day 29, 39 and 49. Mice were sacrificed on day 52 after imaging. (**A**) Quantification of bioluminescence signal (photon/s), data are shown as mean ± SD, *n* = 5. (**B**) Representative images of successive imaging of the same animal in each group, color scale bar indicates photon/s/mm^2^ of the field. (**C**) Correlation of bioluminescent signal with tumor weight for treated (blue dots) and untreated (red dots) mice. Statistical analysis was done by two-way ANOVA with Sidak’s test, *n* = 5 mice were inoculated and treated for each group. ** *p* < 0.01; **** *p* < 0.0001.

To further validate our model with regards to platinum sensitivity and ability to monitor efficacy, OVCAR8^luc^ xenograft-bearing mice were randomized to receive either 30 mg/kg carboplatin or HEPES buffer control and tumor burden was evaluated. Tumor growth was detectable by BLI as early as seven days post-inoculation (Figure 6B). Prior to treatment commencement, no significant differences in BLI signal were observed between groups. Treatments were administered on days 29, 39 and 49 post-tumor inoculation. In controls, BLI demonstrated steady tumor growth throughout the study followed by exponential tumor growth after day 42. Conversely, BLI signal for the treatment group remained at baseline until the endpoint on day 52. Tumor burden in the carboplatin-treated mice was significantly lower compared to the control group, as quantified by bioluminescence signals. Moreover, total tumor weight and volume from the treatment group were significantly lower as compared to controls (Figure S5). In addition, the BLI signal for both control and treatment groups correlated well with tumor burden (*R*^2^ = 0.86, Figure 6C).

## 3. Discussion

Valid and comprehensive animal models are essential for successful translational studies in cancer research. While extensive studies have been conducted on intraperitoneal and subcutaneous xenografts developed from SKOV3 and A2780 cell lines, recent studies have suggested these tumor models may not be representative of HGSOC [13,14]. Indeed, SKOV3 and A2780 cell lines have been shown to form solid tumor nodules with limited dissemination [26]. Given that, in the clinical setting, advanced ovarian cancer presents as multiple small nodules that are spread across the peritoneum, animal models that present with a singular tumor either in the abdominal cavity or the subcutaneous space do not mirror real-life patient situations.

In the present study, we have characterized an OVCAR8^luc^ intraperitoneal xenograft model which presents clinical and pathological features similar to that of HGSOC. Upon dissection, tumor nodules were observed throughout the abdominal cavity up to the diaphragm, often covering and/or obstructing organs such as the large and small intestine, spleen, and kidneys. Given that the gastrointestinal tract and the omentum are reported to be the most common sites for ovarian tumor migration, this pattern of dissemination highlights the clinical similarities to HGSOC. Moreover, immunohistochemical analysis of xenograft samples revealed strong positive staining for known markers of HGSOC such as PAX8 and WT1. The presence of the WT1 signature is particularly interesting due to its prognostic value. Patients with WT1-positive tumors usually present with advanced stage disease and have significantly lower five-year survival rates [27]. Moreover, it has been shown that over-expression of WT1 in SKOV3 cells enhances cell proliferation and invasion [28]. This likely explains the high disseminative feature of OVCAR8^luc^ intraperitoneal tumors, suggesting that the current model may be a valuable tool for investigating novel therapeutics against aggressive and poorly prognostic ovarian cancer.

Our immunodetection and qPCR results demonstrated that BRCA1 deficiency was retained in vivo in OVCAR8^luc^ xenograft. *BRCA* status is an important prognostic factor for ovarian cancer. However, reversion mutations of the *BRCA1/2* genes have been reported both in cell lines [29,30] and in the clinic [31]. Secondary somatic mutation that restored BRCA1/2 has been implicated with resistance to treatment with platinum agents [32,33] and poly(ADP-ribose) polymerase (PARP) inhibitors [34]. Moreover, it has been shown that the most commonly used *BRCA* deficient cell lines such as UWB1.289 and COV362 do not generate peritoneal xenografts [35]. Xenograft samples collected from the presented model maintained a loss in BRCA1 expression, making the current model one of the few intraperitoneal xenograft models with BRCA deficiency. Nevertheless, although BRCA status of patients is an essential stratifying feature that contributes to treatment decisions, patients with germline inactivation of *BRCA1/2* only account for a small portion of the patient population [15]. Instead, over 50% of HGSOC patients exhibited BRCAness through other mechanisms including promoter hypermethylation and somatic mutation in either *BRCA1/2* or other genes involved in the homologous recombination repair pathway (i.e., *CHEK2*, *FANC1*) [16,36]. OVCAR8 has been well characterized to harbor promoter hypermethylation at *BRCA1*. As expected, both the OVCAR8 cell line and the developed xenograft tumors showed high degrees of methylation at the *BRCA1* promoter region. This suggests that the BRCAness phenotype was successfully retained in in vivo xenografts through persistent hypermethylation. Interestingly, significantly higher methylation was observed in the tumor samples as compared to the in vitro cell line. While the reason for this discrepancy remains unknown, similar findings have been previously reported in breast cancer cell lines where increased methylation at the *BRCA1* promoter was observed after inoculation into immunodeficient mice [37].

A key characteristic of BRCA deficiency is in that it confers sensitivity to platinum compounds [38]. It is known that in vitro, *BRCA*-deficient breast cancer cells are sensitive to cisplatin [39]. Furthermore, restoring BRCA1 function results in platinum resistance [40,41]. In ovarian cancer, *BRCA* deficiency due to either mutation or BRCAness syndrome also leads to a heightened sensitivity to platinum agents [42,43,44]. Indeed, we found a dramatic decrease in both bioluminescence signal and tumor burden in the carboplatin treated mice, suggesting that our model demonstrated a high sensitivity to carboplatin.

Bioluminescence was incorporated into this model to monitor disease progression and abdominal invasion in a noninvasive, real-time setting. Due to the disseminated disease presentation, it is not feasible or possible to completely collect all cancerous nodules. Hence, utilizing BLI technology may be a more accurate way to measure tumor burden. Furthermore, the incorporation of BLI allows for ex-vivo imaging as a means to confirm that extracted nodules were obtained from OVCAR8^luc^ cells. To our knowledge, this is the first study to fully characterize a bioluminescent, intraperitoneal xenograft model using OVCAR8 cells. In our initial xenograft model characterization studies, a low to moderate correlation between bioluminescent signal and total tumor weight was observed due to attenuation of bioluminescence signal secondary to the development of ascites at endpoint. In addition, a stronger HIF1-α stain was observed in the 10 million group (Figure S6), suggesting that the relatively lower correlation (*R*^2^ = 0.13) to the 5 million group may be a result of an increased hypoxic condition in conjunction with the attenuation of BLI signal by ascites. As such, we performed the carboplatin efficacy study in mice inoculated with 5 million cells prior to the onset of ascites. In this case, we did not see production of ascites and a strong correlation (*R*^2^ = 0.86) between bioluminescence signal and tumor weight was observed. Similarly, an improved light intensity after ascites drainage was previously reported in a bioluminescent ID8 mouse model [45]. In addition, initiating treatment at low to moderate tumor burden also mimics the current standard of care in the clinic, where chemotherapy is administered to patients after optimal cytoreductive surgery when the tumor and ascites burdens are low. Collectively, the results demonstrate that the current model is valid for early-stage disease; however, caution must be given in interpreting BLI data after the onset of ascites. Nonetheless, the presented model can play an important role for preclinical evaluation of novel therapeutic entities.

## 4. Materials and Methods

### 4.1. Materials

Carboplatin and primer oligo for *BRCA1* promoter and *COL2A* gene were purchased from Sigma Aldrich (Oakville, ON, Canada). RPMI-1640, DMEM media, fetal bovine serum (FBS), penicillin-streptomycin solution, and phosphate buffered saline (PBS) (pH 7.4) were obtained from Life Technologies (Burlington, ON, Canada). RIPA buffer (9806) was purchased from Cell Signaling Technology (Danvers, MA, USA). Primary mouse monoclonal, anti-BRCA1 antibody (sc-6954) was obtained from Santa Cruz Biotechnology (Dallas, TX, USA). Zemo Methylation Lightning Kit (D5030) was purchased from Cedarlane (Burlington, ON, Canada). D-luciferin potassium salt (122799) was obtained from PerkinElmer (Woodbridge, ON, Canada). Primary rabbit monoclonal, anit-HIF1-α antibody (ab51608), primary rabbit monoclonal, anti-Wilms Tumor protein antibody (CAN-R9(IHC)-56-2) were purchased from Abcam (Toronto, ON, Canada). Primary rabbit polyclonal, anti-PAX8 antibody (10336-1-AP) was purchased from Proteintech (Rosemont, IL, USA). FuGENE^®^6 Transfection Reagent (E2692) and pGL4.20[*luc2*/Puro] vector (E6751) were obtained from Promega (Madison, WI, USA).

### 4.2. Cell Line and Transfection of OVCAR8 with Luc2

OVCAR8 was obtained from the Biological Testing Branch of the National Cancer Institute (NCI; Frederick, MD, USA). OVCAR8 was cultured in RPMI-1640 medium supplemented with 10% FBS and penicillin-streptomycin solution to a final concentration of 100 units/mL for penicillin and 100 µg/mL for streptomycin. Cell culture flasks were incubated at 5% CO_2_ at 37 °C with 90% relative humidity. OVCAR8 cells were stably transfected with pGL4.20 plasmid contained modified firefly luciferase (*luc2*) and antibiotic resistance gene for puromycin selection (Puro) using FuGENE^®^6 Transfection Reagent following the manufacturer’s instruction. Briefly, 2 µg of pGL4.20 plasmid was mixed with 100 µL of transfection reagent. OVCAR8 cells cultured with 10 mL of RPMI-1640 media were exposed to the plasmid-transfection reagent mixture for 48 h. Following transfection, cells were selected in RPMI-1640 containing puromycin at a final concentration of 2 µg/mL for one week. The surviving clones (OVCAR8^luc^) were exposed to 1 mM d-luciferin and imaged under Bruker In-Vivo Xtreme II system using Bruker Molecular Imaging software (version 752, Billerica, MA, USA) and subsequently maintained in puromycin-containing media (2 µg/mL).

### 4.3. Animal Studies

In order to develop and characterize an intraperitoneal xenograft model, sub-confluent OVCAR8^luc^ cells were trypsinized and collected by centrifuging at 1000 rpm for 5 min at room temperature. The collected cells were washed with PBS. 5 and 10 million OVCAR8^luc^ cells suspended in 500 µL PBS were inoculated in 8-weeks old female NOD/SCID mice (10 mice per group) via intraperitoneal injection using a 30-gauge needle. The site of injection was massaged immediately after injection to evenly distribute cells. Bioluminescence images were obtained at 2 h post-inoculation for each mouse to establish a baseline (day 0). Tumor growth was monitored by weekly bioluminescence imaging until endpoint. Body weight, abdominal girth, and overall health condition of the mice was monitored daily using body condition scoring. Endpoints were established by the following parameters: hunched posture, failure to groom, >20% weight loss, inactivity, and distended abdomen. Following endpoint, mice were sacrificed for gross evaluation of the disease. Tumor nodules were collected and snap frozen in liquid nitrogen for further analysis. All animal studies were approved by the Office of Research Ethics at the University of Toronto and conducted in accordance with the guidelines of the Canadian Council on Animal Care (protocol number: 20011899, approved 24 April, 2017, renewed 15 May, 2018).

### 4.4. Chemotherapy Treatment

To assess carboplatin sensitivity in our xenograft model, 8-weeks old female NOD/SCID mice were inoculated with 5 million OVCAR8^luc^ cells. Treatment with carboplatin (30 mg/kg in HEPES buffer) via intraperitoneal injection was initiated at day 29 post-inoculation, and doses were repeated on day 39 and day 49. The control group received HEPES buffer. Therapeutic efficacy was investigated by weekly BLI, and animals were sacrificed on day 52 after imaging. Mice were monitored twice daily and body weight loss was used as the main indicator for systematic toxicity. Following post-mortem examination, gross anatomy of tumor location and number of nodules were noted. Tumor nodules collected were weighed and correlated with BLI signal using Pearson’s correlation coefficient.

### 4.5. Bioluminescence Imaging

d-Luciferin (150 mg/kg) was administered via intraperitoneal injection. Mice were then anaesthetized by 2% isoflurane at 2 L/min flow rate in the anesthesia chamber. Subsequently, groups of five mice were moved to an airtight box in the imaging chamber supplied with warm air. Imaging was performed at a distance to allow for whole body imaging. Sequential 2-min exposure for 20 min was performed to ensure the signal was captured at the peak. Following each bioluminescence image, a gray-scale image was also obtained to provide an anatomical reference. Bioluminescence imaging was performed on a Bruker In-Vivo Xtreme II system that consists of a high sensitivity cooled charged-coupled camera mounted in a light-tight chamber. Bruker Molecular Imaging software (version 752, Billerica, MA, USA) was used to acquire and analyze images. For analysis, the region of interest was selected by a threshold value; sum of photon/second was then determined and reported for each time point.

### 4.6. Immunohistochemistry

OVCAR8^luc^ xenograft samples collected at endpoint were fixed in formalin and submitted to Murine Imaging and Histology Core (Toronto, ON, Canada) for processing and paraffin embedding. Paraffin blocks were subsequently sent to Pathology Core at Princess Margaret Hospital (Toronto, ON, Canada) for immunostaining. Briefly, two consecutive sections were sliced onto slides. Slides were dewaxed in xylene followed by passaging through graded alcohol. Sections were immunostained for HIF1-α for assessing the extent of hypoxia, for PAX8 and WT1 for determination of high-grade serous histology, and for Haemotoxylin and Eosin (H&E) staining for overall tissue morphology. Slides were scanned at 20× and 40× magnification by NanoZommer Digital Pathology software (Version 2.7.25, Hamamatzu, Japan).

### 4.7. Western Blotting

BRCA1 protein expression was evaluated in tumor samples as well as in the originating cell line (OVCAR8). The BRCA1 wild type cell line, HEYA8, was also analyzed for comparison of BRCA1 expression. Xenograft samples (50 mg) and cell lysates were mixed with 400 µL of 1× RPIA buffer and homogenized on ice for 20 s. Tissue and cell lysates were incubated on ice and vortexed at maximum speed every 10 min for 1 h, followed by centrifugation at 16,000× *g* for 10 min at 4 °C. Standard procedures were used for immunoblotting as previously described [46]. BRCA1 overexpressed UWB1.289+*BRCA* cell line and a *BRCA1* mutated UWB1.289 cell line was used as positive and negative controls, respectively.

### 4.8. Methylation-Specific Quantitative PCR

The extent of methylation on the *BRCA1* promoter region was investigated using methylation-specific qPCR. HEYA8 was included as a negative control and universal methylated human DNA was used as a positive control. Methylation of original OVCAR8 cells prior to inoculation was used for comparison with the tumor samples. Differential gene amplification by PCR using primer designed specifically for methylated sequence identifies the methylation status. Primer sequences of *BRCA1* promoter for the methylated reaction were 5′ TCG TGG TAA CGG AAA AGC GC 3′ (sense) and 5′ AAA TCT CAA CGA ACT CAC GCC G 3′ (antisense). Methylation-independent primers were designed for *COL2A* gene which served as an internal control; 5′ GGG AAG ATG GGA TAG AAG GGA AT 3′ (sense); 5′ TCT AAC AAT TAT AAA CTC CAA CC 3′ (antisense).

### 4.9. Statistical Analysis

The degree of *BRCA1* promoter methylation was analyzed by one-way ANOVA with Bonferroni’s test. Two-way ANOVA with Sidak’s test was used to investigate the difference in tumor burden (as measured by bioluminescence signal), abdominal girth, and weight loss between treated and untreated mice. Finally, two-tailed, unpaired, student *t*-test with Welch’s correction was done to analyze the difference in tumor weight and tumor volume between control and carboplatin treated mice. For all experiments, a *p*-value < 0.05 was considered statistically significant. All graphs and statistical calculations were performed using GraphPad Prism 6 (version 6.01, San Diego, CA, USA).

## 5. Conclusions

In this study, we developed and characterized a clinically relevant model of BRCA-deficient, HGSOC with histochemical and dissemination patterns that resemble human disease. Importantly, persistent BRCA deficiency, which was attributed to continued hypermethylation of *BRCA1* promoter, provides a valuable tool for investigating novel therapeutics which capitalizes on the synthetic lethality of a non-functional DNA damage repair process. With the strong correlation observed between the bioluminescence signal and tumor burden, the current model may serve as an important preclinical model for ovarian cancer.

## Supplementary Materials

Supplementary materials can be found at https://www.mdpi.com/1422-0067/20/10/2498/s1. Table S1. STR analysis of OVCAR8, OVCAR8^luc^, and subsequent xenograft tumor. Figure S1. Changes in abdominal girth of tumor-bearing mice. Figure S2. Kaplan-Meier Survival curve of tumor-bearing mice without treatment. Figure S3. Tumor growth as quantified by bioluminescence signaling. Figure S4. H&E staining of xenograft revealed necrosis at the tumor core. Figure S5. Effect of carboplatin treatment (30 mg/kg) on tumor weight and volume. Figure S6. Immunostaining of HIF1-α in xenografts from 5 and 10 million groups. Figure S7. Changes in body weight and abdominal girth in mice treated with carboplatin or control.

## Abbreviations

HGSOC	high-grade serous ovarian cancer
BLI	bioluminescent imaging
