# Supplementary material for: Development of a Bioluminescent BRCA1-Deficient Xenograft Model of Disseminated, High-Grade Serous Ovarian Cancer
Source: Int J Mol Sci. 2019 May 21;20(10):2498. doi: 10.3390/ijms20102498 (PMC6566953; doi:10.3390/ijms20102498)
Supplement: Supplementary file 1 [file ijms-20-02498-s001.pdf]

Table S1. STR analysis of OVCAR8, OVCAR8^{luc} cell lines and subsequent xenograft tumor samples from mice 96e, 97L, 99d and 99e. 97L-m and 99e-m represent samples from metastatic lesions isolated from small intestine.

		STR analysis result		Reference profile	
**Cell line**	**Marker**	Allele 1	Allele 2	Allele 1	Allele 2
OVCAR8	AMEL	X	X	X	X
	CSF1PO	11	11	11	11
	D13S317	12	12	12	12
	D16S539	13	13	13	13
	D21S11	28	28	28	28
	D5S818	12	12	12	12
	D7S820	12	12	12	12
	TH01	7	7	7	7
	TPOX	8	8	8	8
	vWA	16	17	16	17
OVCAR8^{luc}	AMEL	X	X	X	X
	CSF1PO	11	11	11	11
	D13S317	12	12	12	12
	D16S539	13	13	13	13
	D21S11	28	28	28	28
	D5S818	12	12	12	12
	D7S820	12	12	12	12
	TH01	7	7	7	7
	TPOX	8	8	8	8
	vWA	16	17	16	17
96e	AMEL	X	X	X	X
	CSF1PO	11	11	11	11
	D13S317	12	12	12	12
	D16S539	13	13	13	13
	D21S11	28	28	28	28
	D5S818	12	12	12	12
	D7S820	12	12	12	12
	TH01	7	7	7	7
	TPOX	8	8	8	8
	vWA	16	17	16	17
97L	AMEL	X	X	X	X
	CSF1PO	11	11	11	11
	D13S317	12	12	12	12
	D16S539	13	13	13	13
	D21S11	28	28	28	28
	D5S818	12	12	12	12
	D7S820	12	12	12	12
	TH01	7	7	7	7
	TPOX	8	8	8	8
	vWA	16	17	16	17
97L-m	AMEL	X	X	X	X
	CSF1PO	11	11	11	11

	D13S317	12	12	12	12
	D16S539	13	13	13	13
	D21S11	28	28	28	28
	D5S818	12	12	12	12
	D7S820	12	12	12	12
	TH01	7	7	7	7
	TPOX	8	8	8	8
	vWA	16	17	16	17
99d	AMEL	X	X	X	X
	CSF1PO	11	11	11	11
	D13S317	12	12	12	12
	D16S539	13	13	13	13
	D21S11	28	28	28	28
	D5S818	12	12	12	12
	D7S820	12	12	12	12
	TH01	7	7	7	7
	TPOX	8	8	8	8
	vWA	16	17	16	17
99e	AMEL	X	X	X	X
	CSF1PO	11	11	11	11
	D13S317	12	12	12	12
	D16S539	13	13	13	13
	D21S11	28	28	28	28
	D5S818	12	12	12	12
	D7S820	12	12	12	12
	TH01	7	7	7	7
	TPOX	8	8	8	8
	vWA	16	17	16	17
99e-m	AMEL	v	X	X	X
	CSF1PO	11	11	11	11
	D13S317	12	12	12	12
	D16S539	13	13	13	13
	D21S11	28	28	28	28
	D5S818	12	12	12	12
	D7S820	12	12	12	12
	TH01	7	7	7	7
	TPOX	8	8	8	8
	vWA	16	17	16	17

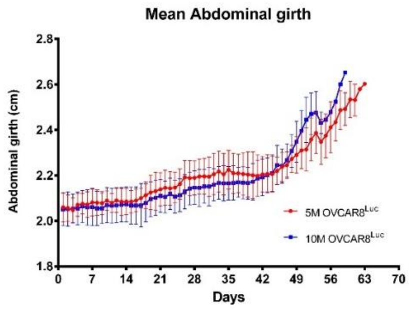

Figure S1 Changes in abdominal girth of tumor bearing mice. Data are shown as mean ± SD, $n = 8$ for mice inoculated with 5 million cells, $n = 9$ for mice inoculated with 10 million cells.

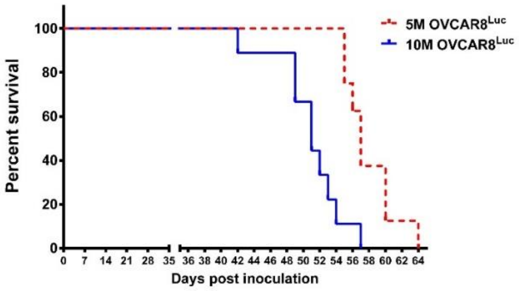

Figure S2. Kaplan-Meier Survival curve of tumor bearing mice without treatment. Statistical analysis was done by Mantel-Cox test, $p = 0.005$, $n = 8$ for mice inoculated with 5 million cells, $n= 9$ for mice inoculated with 10 million cells.

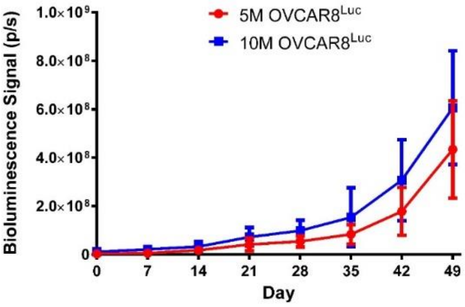

Figure S3. Tumor growth as quantified by bioluminescence signal. Data are shown as mean ± SD. Statistical analysis was done by two-way ANOVA with Sidak's test. $n = 8$ for mice inoculated with 5 million cells, $n = 9$ for mice inoculated with 10 million cells.

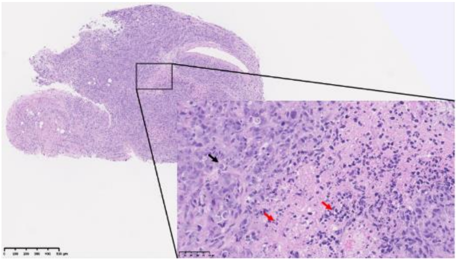

Figure S4. H and E staining of xenograft revealed necrosis at the tumor core. The figure shows a representative image of a xenograft sample at 4× magnification and the insert represents 40× magnification. The black arrow indicates normal cells with intact nucleus and cytoplasm. The red arrows indicate compacted nuclei indicative of cell death. Scale bar equal to 500 and 50 μm for 4× and 40× magnification, respectively.

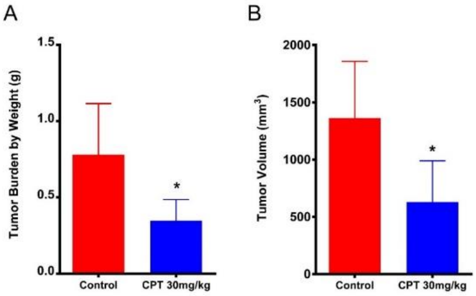

Figure S5. Carboplatin (30 mg/kg) treatment significantly reduce (**A**) tumor weight and (**B**) tumor volume. Statistical analysis was done by two-tailed, unpaired student t-test with Welch's correction, $n = 5$ for each group. * $p < 0.05$.

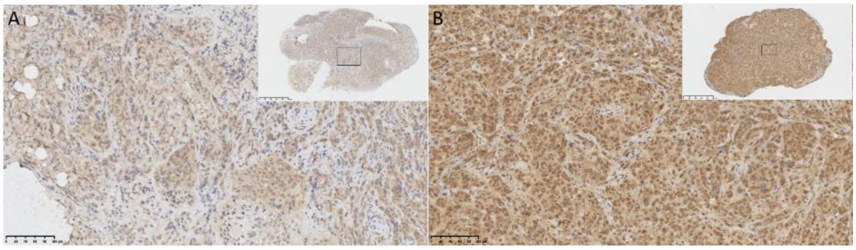

Figure S6. Immunostaining of HIF1-α in xenograft obtained from mouse inoculated with (**A**) 5 million and (**B**) 10 million OVCAR8^{luc} cells. The figure shows a representative image of a xenograft sample at 4× magnification and the insert represents 40× magnification. Scale bar equal to 500 and 50 μm for 4× and 40× magnification, respectively.

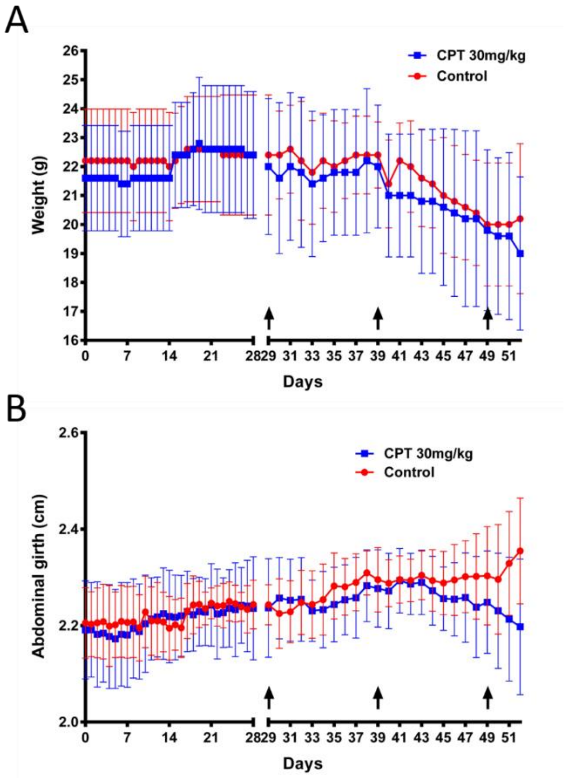

Figure S7. Changes in (**A**) body weight and (**B**) abdominal girth in tumor bearing mice receiving control or carboplatin treatment. Data are shown as mean ± SD. Statistical analysis was done by two-way ANOVA with Sidak's test, n = 5 for each group. However, no significance was found. Black arrow indicates treatment days (day 29, 39 and 49 post-inoculation).
